# Survival of dental implants in patients with oral cancer treated by surgery and radiotherapy: a retrospective study

**DOI:** 10.1186/1472-6831-15-5

**Published:** 2015-01-20

**Authors:** Giorgio Pompa, Matteo Saccucci, Gabriele Di Carlo, Edoardo Brauner, Valentino Valentini, Stefano Di Carlo, Tina Gentile, Giorgio Guarino, Antonella Polimeni

**Affiliations:** Department of Oral and Maxillofacial Science, Sapienza University of Rome, Via Caserta 272/A, Rome, Italy

**Keywords:** Dental implants, Osseointegration, Radiation

## Abstract

**Background:**

The aim of this retrospective study was to evaluate the survival of dental implants placed after ablative surgery, in patients affected by oral cancer treated with or without radiotherapy.

**Methods:**

We collected data for 34 subjects (22 females, 12 males; mean age: 51 ± 19) with malignant oral tumors who had been treated with ablative surgery and received dental implant rehabilitation between 2007 and 2012. Postoperative radiation therapy (less than 50 Gy) was delivered before implant placement in 12 patients. A total of 144 titanium implants were placed, at a minimum interval of 12 months, in irradiated and non-irradiated residual bone.

**Results:**

Implant loss was dependent on the position and location of the implants (P = 0.05–0.1). Moreover, implant survival was dependent on whether the patient had received radiotherapy. This result was highly statistically significant (P < 0.01). Whether the implant was loaded is another highly significant (P < 0.01) factor determining survival. We observed significantly better outcomes when the implant was not loaded until at least 6 months after placement.

**Conclusions:**

Although the retrospective design of this study could be affected by selection and information biases, we conclude that a delayed loading protocol will give the best chance of implant osseointegration, stability and, ultimately, effective dental rehabilitation.

## Background

Head-and-neck cancer is the most common cancer worldwide with an estimated global incidence of 500,000 new cases annually, three-quarters of which are in underdeveloped countries. The vast majority (approximately 90%) of head-and-neck cancers are squamous cell carcinomas [1]. According to a recent review, in the United States the 5-year survival rate of head and neck cancer is 57% [2]. Patients with oral cancer are commonly treated by a combination of radiotherapy and ablative surgery. After radical cancer surgery, the oral rehabilitation of a patient is a demanding procedure. Following radiation and surgical resection, most patients suffer from soft and hard tissue defects resulting in functional disabilities and esthetic deformity [3]. Dental rehabilitation using conventional prostheses may be compromised or precluded by disadvantageous changes in oral anatomy, and radiotherapy can produce mucositis, xerostomia and disruption of bone healing processes [3]. In this situation, dental implants can potentially result in a more effective oral rehabilitation in terms of mastication, esthetics and speech function. However, even implant treatment in oral cancer patients is challenging because the bone into which the dental implants are placed has often been within the field of irradiation, or is grafted. Implant failure increases when they are placed in irradiated bone [3], in part because radiotherapy can result in progressive fibrosis of vessels and soft tissue, leading to diminished healing capacity. In addition radiations impedes the osseointegration of implants by reducing bone vascularity, clinically expressed as osteoradionecrosis. The interaction between ionizing radiation and tissue causes damage to the bone, periosteum, and connective tissue of the mucosa and the endothelium of the vessels, which at later stages leads to hypoxia, hypocellularity and hypovascularity in the affected tissues, and the loss of resistance against infection and trauma [4, 5]. Tissue dehiscence and osteoradionecrosis can occur, and often leads to implant loss. Implant treatment of irradiated patients is dependent upon issues like the timing of implant placement in relation to the radiation therapy, the anatomic site chosen for implant placement, the radiation dosage at that site and the consequent risk of osteoradionecrosis [6, 7]. The aim of the present study, we evaluate the survival of dental implants in patients affected by oral cancer, treated with surgical and radiotherapy.

## Methods

**Figure 1:**
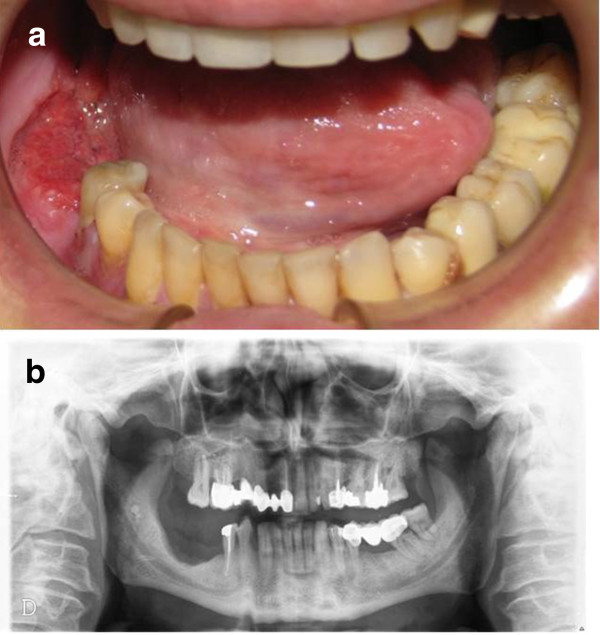
**Patient affected by squamous cell carcinoma. a)** intraoral image of the squamous cell carcinoma, **b)** ortopanoramic radiograph after ablative surgery.

**Figure 2:**
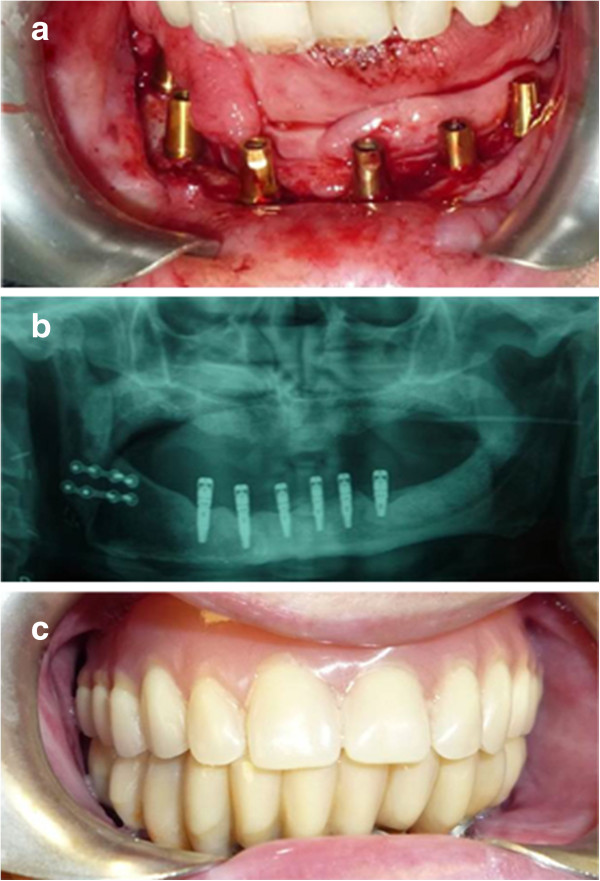
**Patient after implant-supported prosthesis rehabilitation. a)** implants placed in the jaw after revasculated flap reconstruction, **b)** final panoramic radiograph with ossointegrated implants, **c)** implant-supported prosthesis.

This study was conducted as a retrospective study at the Department of Oral and Maxillofacial Science, Sapienza University of Rome. Data were collected for a period between 2007 and 2012. The study comprised 34 subjects (22 females, 12 males) with malignant oral tumors (22 in the mandible/floor of the mouth, 12 in the maxilla) who underwent dental implant rehabilitation. The patients had undergone ablative surgery with or without adjunctive radiation therapy. The study was approved by the Ethics Committee at “Sapienza“ University of Rome (ref. n° 3452). All subjects gave their signed informed consent to medical and surgical procedure and to the use of data in this research. The mean age of the patients at the time of surgery was 51 ± 19 years. Patients with certain systemic disease (uncontrolled diabetes mellitus) and smokers were excluded [8]. The most prevalent tumor diagnosed was squamous cell carcinoma (n = 16). Other tumor types were: ameloblastoma (n = 6); osteosarcoma (n = 4); pleomorphic adenoma (n = 4); fibrous dysplasia (n = 2); and nasopharyngeal angiofibroma (n = 2). Orofacial defects in 26 patients were reconstructed microsurgically using a range of revasculated flap techniques (Figure 1). A total of 168 titanium implants were placed in irradiated or non-irradiated residual bone, with a minimum interval of 12 months between irradiation and implant placement. This procedure was performed by an experienced oral surgeon (G.P). The minimum implant length was 10 mm. In this study, indirectly irradiated bone was considered as non-irradiated. The time interval between radical oral cancer surgery, radiation therapy and implant placement, respectively, ranged from 12–89 months. After implant placement patients underwent to a routine follow-up at 1 month, 3 months, 6 and 12 months performing an intraoral radiograph. *.* Based on the histological findings, post-operative radiotherapy was set in accordance with the NCCN guidelines [9] *.* Considering this, postoperative radiation therapy (less than 50 Gy) was delivered before implant placement to 12 patients and was delivered in fractions of 2 Gy given daily for 5 days each week (Table 1). The OSSEOTITE® implants (3i Biomet, Palm Beach Gardens, FL, USA) were made from commercially pure titanium (grade IV) treated with a specific, proprietary dual acid-etching protocol. The acid-etch protocol does not include the coronal 3 mm of the titanium surface, which is machined instead. All implants (n = 168) were placed in jaws affected by surgical resection. There were 152 and 16 implants inserted into non irradiated and irradiated bone, respectively. The implant primary stability was evaluated measuring the torque at the moment of insertion. The torque level was not superior of 40 Nm. We have planned to use these implants as abutments for removable overdentures (12 patients), screw-retained fixed dentures (11 patients) and cement-retained implant-supported prostheses (11 patients). Implant survival was evaluated within five different subgroups: location (maxilla *vs*. mandible); implant site (anterior *vs*. posterior); gender (male *vs*. female); radiotherapy (irradiated *vs*. non-irradiated); and time after initial loading (immediate, <6 months, 6 months, and >6 months) (Figure 2). Regarding the perio conditions, patients were followed by an Oral Hygienist before surgical procedures, where the patients received instructions to maintain oral health and care. The dental elements parodontally compromised were extracted as well. After surgery an Oral Hygiene follow up was performed every 3 months in the first year after implantation and every 6 months thereafter. Patients were evaluated at each review by clinical and radiographic examination. Implants were considered to be successful when there were no patient complaints, implant mobility, peri-implantitis [7]. Survival time was measured from initial implantation to either the failure (removal) or the last review of the implant. In the present study, we considered aesthetics and functional characteristics for each patient to optimize the results of the final implant-prosthetic rehabilitation. We used patients’ existing removable partial or complete dentures as templates for implant planning. As a consequence, our implant treatment was “prosthetically driven”.

**Table 1:** **Patients and radiation characteristics**

Patient	Radiotherapy dose (Gy)	Standard fractionation therapy (2 Gy daily)/Hyperfractioned therapy
1	50	Standard fractionation therapy
2	50	Standard fractionation therapy
3	44	Standard fractionation therapy
4	48	Standard fractionation therapy
5	48	Standard fractionation therapy
6	44	Standard fractionation therapy
7	48	Standard fractionation therapy
8	44	Standard fractionation therapy
9	50	Standard fractionation therapy
10	48	Standard fractionation therapy
11	40	Standard fractionation therapy
12	50	Standard fractionation therapy

The observation mean period after radiation therapy was 39,5 months with a standard deviation of 22,8 months. Moreover, the mean observation period after implant placement is 22,9 months with a SD of 15.5 months.

### Statistical analysis

Implant survival was evaluated within the subgroups listed above. The null hypothesis of independence between implant survival and the various factors were tested by Pearson’s chi-squared test [10]. We also used Fisher’s Exact Test [11] for count data, as the chi-squared test can be misleading when the frequency in a single cell is less than 5 units.

## Results

Sixteen implants (9.5%) failed over the study period: six during the healing phase, two during the loading phase and eight due to recurrence of the cancer. In the mandible, 96 implants were inserted: 28 anteriorly and 68 posteriorly. In the maxilla, 72 implants were inserted: 26 anteriorly and 46 posteriorly. In 34 patients, the initial prosthodontic treatment was maintained throughout the observation period. Two patients had recurrence of their cancer, which required a second surgical intervention during which all eight mandibular implants were removed. Dehiscence with disturbed wound healing occurred in four irradiated patients, while dehiscence and oroantral communication was observed in two irradiated patients.

The frequency and distribution of implant lost among the population is shown in Table 2.

**Table 2:** **Frequency and distribution of implant lost among the population**

	Less 30	30 - 60	More 60	Total
Frequency				
F	0	0	10	10
M	0	2	4	6
Total	0	2	14	16
Percentage				
F	0,0%	0,0%	62,5%	62,5%
M	0,0%	12,5%	25,0%	37,5%
Total	0,0%	12,5%	87,5%	100,0%

Descriptive analysis of these factors is shown in Tables 3, 4, 5 and 6. Overall, implant survival was not dependent on whether the implant was placed in the maxilla or mandible (Table 3; p > 0.1). However, there was a significant correlation between the rate of mandibular implant loss and implant position, with all failing implants being in a posterior position (Table 3; p = 0.05–0.1). In contrast, there was no correlation in either the maxilla or the mandible between an implant surviving and its placement position (Table 4; p > 0.8). Furthermore, implant survival was significantly linked with radiotherapy (p < 0.01): implants in non-irradiated bone predominantly survived, whereas more implants in irradiated bone failed (Table 5). Finally, implant survival is highly dependent on the interval period before loading. (Table 6; p < 0.01).

**Table 3:** **Implants by survival (yes or no) and location (mandible, maxilla), Odds ratio: 0.4137915, p-value = 0.1845**

	Mandible	Maxilla	Total
Yes	84 (50,0%)	68 (40,5%)	152 (90,5%)
No	12 (7,1%)	4 (2,4%)	16 (9,5%)
Total	96 (57,1%)	72 (42,9%)	168 (100,0%)
Anterior	0	2	2
Posterior	12	2	14
Total	12	4	16

Odds ratio: 0.4137915, p-value = 0.1845.

Below, lost implants by location (mandible, maxilla) and position (anterior, posterior). Odds ratio = 0 p-value = 0.05.

**Table 4:** **Survived implants by location (mandible, maxilla) and position (anterior, posterior)**

	Anterior	Posterior	Total
Mandible	28	56	84
Maxilla	24	44	68
Total	52	100	152
	**Anterior**	**Posterior**	**Total**
Mandible	18,40%	36,80%	55,30%
Maxilla	15,80%	28,90%	44,70%
Total	34,20%	65,80%	100,00%

Odds ratio: 0.9171932 p-value = 0.8641.

**Table 5:** **Implants by survival (yes or no) and radiotherapy (not irradiated, irradiated bone)**

	Not irradiated	Irradiated	Total
Yes	113	39	152
No	4	12	16
Total	117	51	168
	**Not irradiated**	**Irradiated**	**Total**
Yes	67,30%	23,20%	90,50%
No	2,40%	7,10%	9,50%
Total	69,60%	30,40%	100,00%

Odds ratio: 8.561084 p-value = 0.0001423.

**Table 6:** **Implants by survival (yes or no) and number of implants loaded at different time (immediate, less than 6 months, 6 months, more than 6 months)**

	Immediate	Less than 6 months	6 months	More than 6 months	Total
Yes	44	17	52	39	152
No	4	8	4	0	16
Total	48	25	56	39	168
	**Immediate**	**Less than 6 months**	**6 months**	**More than 6 months**	**Total**
Yes	26,20%	10,10%	31,00%	23,20%	90,50%
No	2,40%	4,80%	2,40%	0,00%	9,50%
Total	28,60%	14,90%	33,30%	23,20%	100,00%

## Discussion

Dental implants play a crucial role in the therapy of patients affected by malignancies in the head-and-neck region. The goal of implant rehabilitation is to improve the quality of life of these patients by allowing proper retention of removable prostheses and a reduction in the load placed on vulnerable soft tissues [12]. Several factors influence implant survival, especially when patients undergo surgical removal of the malignancies. Indeed, the experience of the surgeon, bone quality, and technical aspects such as implant length, diameter and primary stability each play pivotal roles. After oral cancer surgery, additional factors influence implant osseointegration, such as bone topography and applied radiation dose [13]. Moreover, poor general health, diminished oral hygiene, smoking and alcohol abuse all reduce implant survival.

Radiation therapy is often the first line of therapy for patients with head and neck cancer and is often used as an adjunct to surgical excision. There are three different types of radiotherapy: external beam radiation, brachytherapy, and radioisotope therapy. For the treatment of head-and-neck cancer, external beam methods are most commonly used [13]. Radiation guidelines are variable depending on the method of radiotherapy selected as well as the type, location, and stage of the cancer. Therapeutic radiation protocols for head-and-neck tumors commonly consist of 50–70 Gy [14]. Usually, the radiation dose is given in fractions of ≈ 2 Gy given either once a day (standard fractionation therapy) or twice a day (hyperfractionated therapy) for a defined time period. According to Anderson *et al.*, fractions can be administered every day for 25 days or for 5 days a week for a 5–7-week period [15].

Our results indicate that implant survival is strongly influenced by radiotherapy, confirming previous findings [16] demonstrating that radiotherapy is an important factor in implant failure. Ihde *et al.* [17] report that implant failure is a more significant risk (up to 12 times greater) in irradiated bone rather than in non-irradiated bone. Yerit *et al.* [4], using a comparable protocol of irradiation to that used here, found that mandibular implants were significantly less likely to survive in irradiated bone than in non-irradiated bone.

Although our findings indicate that radiotherapy is an important factor in implant failure, the impact of the position of implant placement within irradiated bone remains contentious. There is much variation in the reported success rates of implant rehabilitation. Recently, De La Plata *et al*. [3] reported that the overall 5-year survival rate in irradiated patients was 92.6%, although irradiated patients had a slightly but significantly higher rate of implant loss than non-irradiated patients. Linsen *et al*. [13] reported implant success rates of over 89% in irradiated bone at 1-, 5-, and 10-year follow-up. However, although these findings suggest a smaller influence of radiation on implant survival than we found here, this discrepancy may be explained by differences between their radiation protocol and our own.

Dosage protocol is a crucial factor with regard to radiation therapy. There is no literature consensus regarding the radiation dosage at which implants begin to experience increased risk of failure. Indeed, Javed *et al*. [18] observed that dental implants showed up to 100% osseointegration when exposed to radiation dosages up to 65 Gy, and suggested that radiation dosages between 50–65 Gy do not negatively influence osseointegration. Conversely, several authors [4, 19, 20] concluded that a total dose less than 50 Gy is necessary to minimize the negative effects of radiotherapy. In conclusion, it seems realistic to assume that full-course radiotherapy (50–65 Gy) is not an absolute contraindication to implant surgery, but that determination of the absolute risk of implant survival must take into account the other contributory factors, as described here.

The optimal timing of implant placement in radiotherapy patients is controversial [21]. Some authors recommend the insertion of implants following the ablative procedure [21–24]. This is advantageous because initial implant healing (osseointegration) takes place before irradiation and there is a reduced risk of late complications, such as the development of osteoradionecrosis [20–22]. However, there is a risk of inappropriate implant positioning, which makes subsequent prosthodontic treatment more complex [13, 18, 19]. There is also a risk that early tumor recurrence will negate the benefits of the implant therapy [25]. In this study, we opted for delayed implant insertion to avoid these complications. However, there is also little consensus on the optimal time interval between irradiation and implant placement. Although implant placement is performed generally no earlier than 6 months after irradiation, Ganström *et al*. suggest that implant therapy should be complete by 6–18 months after radiation [19]. However, Sammartino *et al*. [8] recommend waiting at least 12 months to achieve the best clinical results. It is important to note that the risk of osteoradionecrosis after radiotherapy in head-and-neck cancer patients does not diminish over time because it is underpinned by the progressive and irreversible loss of capillaries [16]. Moreover, immediate implant insertion can be problematic because ablative surgery alters bone anatomy extensively. In our study, the time interval from radical oral cancer surgery through radiation therapy until implant placement ranged from 11–89 months (mean: 39.58).

For the rehabilitative implant-supported prosthesis, we aimed to achieve group function as an occlusal scheme and no mucosal contact, to minimize the risk of mucosal complications. The reduction of mucosal contact is important because fragile mucosa and severe mucositis are common manifestations observed long after radiation therapy. This increases the risk that prosthetic pressure lesions will result in septic osteoradionecrosis [26, 27].

There was a highly significant relationship between the time of loading and the success of the implant in irradiated bone. Good results were obtained with an implant loading protocol of 6 months, and no implants were lost when the period of healing was greater than 6 months. These data support those of Dholam *et al*. [28], who commented on the importance of bone healing and the slow rate of osseointegration in irradiated bone. These data thus do not support immediate loading [28], and we would recommend a period of 6 months or more before loading implants in irradiated bone.

We found no relationship between implant survival and the location of placement (maxilla *vs*. mandible). We did find a stark discrepancy in implant failure in terms of positioning. Implants in the posterior mandible were much more frequently lost than in other positions. This finding is in disagreement with the conclusions of a recent review [20] that reported better outcomes in the mandible. We are unclear why this contradiction occurred. Regarding the influence of prosthetic rehabilitation on implants survivals, it is important to underline that no standard prosthetic appliance is described in the literature. This is due to the large interindividual variability regarding the topography and dimension of the defects. Nevertheless, in this study as well as in a previous study [8] no specific superstructure was found to be particularly favorable in terms of implant survival.

## Conclusions

Irradiated bone is a challenging environment for implant placement. Successful rehabilitation of irradiated patients with implant-supported prostheses is multifactorial. Although the retrospective design of this study could be affected by selection and information biases our results, leads us to believe that immediate [29] and progressive [30] loading protocols are not advisable in irradiated patients. We conclude that a delayed loading protocol will give the best chance of implant osseointegration, stability and, ultimately, effective dental rehabilitation.
